# The influence of biogeographic history on the functional and phylogenetic diversity of passerine birds in savannas and forests of the Brazilian Amazon

**DOI:** 10.1002/ece3.3904

**Published:** 2018-03-03

**Authors:** Sara Miranda Almeida, Leandro Juen, Fernando Landa Sobral, Marcos Pérsio Dantas Santos

**Affiliations:** ^1^ Programa de Pós‐graduação em Zoologia Universidade Federal do Pará Belém Pará Brazil; ^2^ Instituto de Ciências Biológicas Universidade Federal do Pará Belém Pará Brazil; ^3^ Departamento de Ecologia Instituto de Ciências Biológicas Universidade Federal de Goiás Goiânia Goiás Brazil

**Keywords:** bird assemblages, community phylogenetics, ecological traits, functional beta diversity, functional biogeography, open vegetation, phylogenetic relationship

## Abstract

Passeriformes is the largest and most diverse avian order in the world and comprises the Passeri and Tyranni suborders. These suborders constitute a monophyletic group, but differ in their ecology and history of occupation of South America. We investigated the influence of biogeographic history on functional and phylogenetic diversities of Passeri and Tyranni in forest and savanna habitats in the Brazilian Amazon. We compiled species composition data for 34 Passeriformes assemblages, 12 in savannas and 22 in forests. We calculated the functional (Rao's quadratic entropy, FD_*Q*_) and phylogenetic diversities (mean pairwise distance, MPD, and mean nearest taxon distance, MNTD), and the functional beta diversity to investigate the potential role of biogeographic history in shaping ecological traits and species lineages of both suborders. The functional diversity of Passeri was higher than for Tyranni in both habitats. The MPD for Tyranni was higher than for Passeri in forests; however, there was no difference between the suborders in savannas. In savannas, Passeri presented higher MNTD than Tyranni, while in forest areas, Tyranni assemblages showed higher MNTD than Passeri. We found a high functional turnover (~75%) between Passeri and Tyranni in both habitats. The high functional diversity of Passeri in both habitats is due to the high diversity of ecological traits exhibited by species of this group, which enables the exploitation of a wide variety of resources and foraging strategies. The higher Tyranni MPD and MNTD in forests is likely due to Tyranni being older settlers in this habitat, resulting in the emergence and persistence of more lineages. The higher Passeri MNTD in savannas can be explained by the existence of a larger number of different Passeri lineages adapted to this severe habitat. The high functional turnover between the suborders in both habitats suggests an ecological strategy to avoid niche overlap.

## 1 INTRODUCTION

Biological assemblages are the result of both contemporary ecological processes (Chase & Leibold, 2003; Hutchinson, 1957; Weiher & Keddy, 1999) and biogeographic history (Ackerly, 2003; Haffer, 1978; Tofts & Silvertown, 2000). To understand the distribution patterns of biological diversity, ecologists have developed innovative tools that enable them to capture diversity gradients and infer their causes. A recent and promising approach is functional biogeography, which studies the geographical distribution of functional and phylogenetic diversity of assemblages to help explain biological diversity gradients (Violle, Reicc, Pacala, Enquist, & Kattge, 2014). Functional diversity is a measure that quantifies the differences between species by means of ecological traits that affect their fitness and that respond to the biotic and abiotic factors of the environment (Petchey & Gaston, 2006; Tilman, 2001). Phylogenetic diversity, in turn, quantifies the relationships of kinship between species, capturing the evolutionary history of assemblages (Gerhold, Cahill, Winter, Bartish, & Prinzing, 2015; Webb, Ackerly, McPeek, & Donoghue, 2002).

Biogeographic history has an important influence on species diversity gradients (Kennedy et al., 2014) and on the structure of biological assemblages at larger spatial scales (Dreiss et al., 2015; Ma, Sandel, & Svenning, 2016). Biogeographic events (e.g., speciation, dispersion) affect regional diversity (Cracraft, 1994), determining which species may occupy a given biome, habitat, or local assemblage. (Duarte, Bergamin, Marcilio‐Silva, Seger, & Marques, 2014; Ma et al., 2016). For instance, the geographical distribution of different taxa depends on historical processes promoting the dispersal of species into a region, *in situ* speciation (Cavender‐Bares, Kozak, Fine, & Kembel, 2009; Wiens & Donoghue, 2004) , and directly influencing the potential colonizing clades (Jønsson, Lessard, & Ricklefs, 2015).

Ecological processes operating at smaller spatial scales (e.g., different types of environmental filters, differences in resource quantity between habitat types) determine the species composition of local assemblages (Hughes & Eastwood, 2006; Lamarre et al., 2016). For example, habitats with high environmental heterogeneity, high productivity, and niche availability allow the colonization and establishment of many clades (lineages) and species with different ecological traits (Dreiss et al., 2015; Hurlbert & Jetz, 2010). In contrast, habitats with severe environmental conditions, low environmental heterogeneity, and few available niches and resources may select only a few clades with similar ecological traits for colonization and settlement (Dreiss et al., 2015; Gianuca, Dias, Debastiani, & Duarte, 2014; Weiher & Keddy, 1999).

The Amazon is environmentally heterogeneous (Tuomisto et al., 1995), consisting of periodically flooded regions (e.g., varzeas and igapós) and nonflooded regions (*terra firme* forests and savannas). About 80% of the Brazilian Amazon is formed by *terra firme* forests (Pires & Prance, 1985), which contain large trees (23–45 m), high local diversity, and high variation in composition, distribution, and density of plant species between sites (Lima Filho et al., 2001; Oliveira & Mori, 1999; Pitman et al., 2001). Savanna occupies three to four percent of this region and comprises areas of different sizes inserted within a matrix of forest habitats (Pires & Prance, 1985). Unlike forests, savannas are more open with a mosaic of grasses, shrubs, and sparse small trees (Haffer, 1969; Silva & Bates, 2002). These open areas are frequently subject to natural disturbances such as fire and drought, while forests are less disturbed and more stable and productive (Furley, 2006).

The proportion of savanna and forest is historically variable. During the Tertiary and Quaternary periods, these biomes changed their distribution and fragmented into isolated forest patches, expanding and rejoining according to the climatic conditions. Thus, during the dry periods savannas dominated the Amazonian landscape, while forests persisted in large patches called refuges (Haffer, 1969; Sarmiento, 1983). Passeriformes (passerine birds) is the largest and most diverse avian order in the world, representing almost 60% of all living birds. Due to their widespread distribution and great diversity, passerine birds have been the focus of many ecological (Ricklefs, 2002) and evolutionary (Ericson, Klopfstein, Irestedt, Nguyen, & Nylander, 2014; Kennedy et al., 2014) studies. This order comprises a monophyletic group that is divided into two suborders: Passeri (or Oscines) and Tyranni (or Suboscines) (Prum et al., 2015; Sibley & Ahlquist, 1990). Both originated in southern Gondwana, but had different routes of dispersal in the New World (Barker, Cibois, Schikler, Feinstein, & Cracraft, 2004; Boles, 1995; Claramunt & Cracraft, 2015). Tyranni are numerically dominant in South America due to the long period that this continent remained isolated from others. Passeri dominated the other continents, and dispersal to South America seems to have been facilited by the formation of the Isthmus of Panama, which connected North and South America about 3 million years ago (O'Dea et al., 2016; Vuilleumier, 1985). This connection allowed avian lineages from the northern Nearctic regions (e.g., Passeri) to invade the tropics and radiate throughout South America. However, species with South American tropical origins (e.g., Tyranni) remain largely restricted to Neotropical regions (Smith & Klicka, 2010).

Passeri occupy tropical and temperate regions, while Tyranni are more restricted to tropical and subtropical regions (Feduccia, 1999; Newton, 2003; Swanson & Bozinovic, 2011). In South America, Passeri are predominantly found in the forest canopy and open landscapes, while Tyranni have primarily diversified in the forest understory (Ricklefs, 2002; Slud, 1976). Passeri differ from Tyranni in many ecological traits, namely Passeri have a dispersal capacity which allows long distance flights and possess greater flexibility in habitat use, while many Tyranni are restricted to the forest interior and have low dispersal capacity (Moore, Robinson, Lovette, & Robinson, 2008; Weir, Berminghamb, & Shluter, 2009). It has been suggested that these differences are some of the key factors determining the current distributions of these two suborders (Kennedy et al., 2014) and that they also contributed to the high diversification rates recorded for Passeri after their entry into South America (Barker, Burns, Klicka, Lanyon, & Lovette, 2013; Kennedy et al., 2014; Ricklefs, 2002).

The different ecological characteristics and biogeographic histories of the Passeriformes suborders may have generated distinct patterns of functional and phylogenetic diversity. In order to evaluate the importance of the colonization history of forests and savannas by Passeriformes and habitat structure in regard to the diversity of assemblages, we analyzed the diversity measures of Passeri and Tyranni in these two environments. Therefore, we tested the following predictions: (1) In forest areas, Tyranni assemblages should reveal higher functional and phylogenetic diversity than Passeri due to the long period of colonization of this habitat by Tyranni. (2) In savannas, Passeri assemblages should be functionally and phylogenetically more diverses than Tyranni as Passeri have greater flexibility in habitat use, which may have allowed greater success in niche occupation and opportunities for speciation. (3) Passeri assemblages should show a higher diversity of more recent lineages than Tyranni in forest and savanna habitats as Passeri colonized South America more recently than Tyranni.

Finally, because the ecological traits of the species are closely and strongly linked to the resources used within each habitat type, we evaluated the functional beta diversity between the Passeri and Tyranni suborders in both forest and savanna areas. We also identified which ecological traits of Passeri and Tyranni were most associated with forest and savanna habitats. We expected to find higher functional turnover between Passeri and Tyranni in forest areas than in savanna areas. Forests present greater availability and variety of niches than savannas and, therefore, the two suborders potentially perform more distinct ecological functions within forests.

## 2 MATERIAL AND METHODS

### 2.1 Species occurrence data

**Figure 1:**
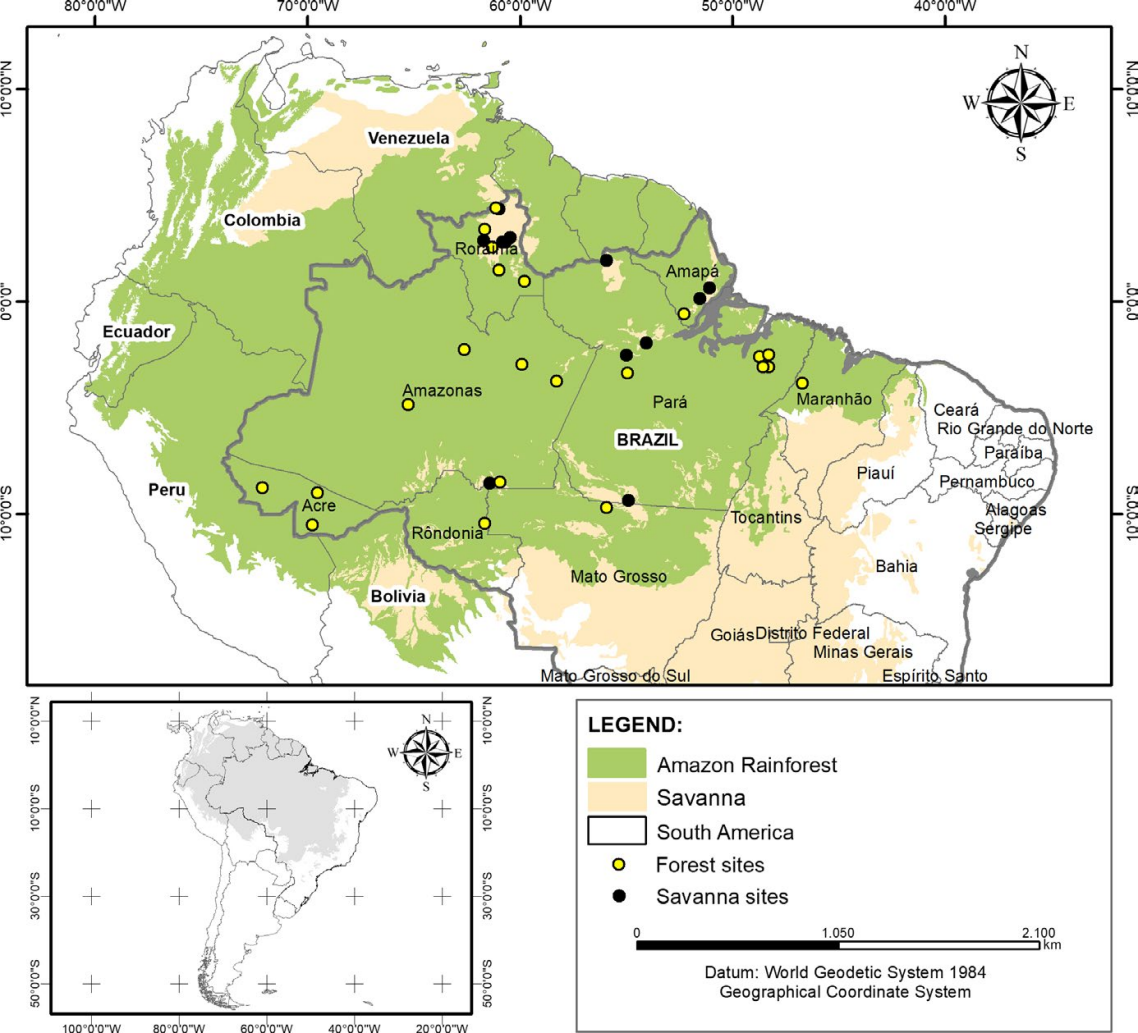
Location of 34 Passeriformes assemblages compiled from studies carried out in 22 forest sites (yellow dots) and 12 savanna sites (black dots), all within the Brazilian Amazon

Based on information in published literature, we compiled data on bird species composition occurring in 34 locations within the Brazilian Amazon, of which 22 were in *terra firme* forest areas and 12 in savanna areas (Figure 1). We obtained information on the geographical coordinates (see Appendix S1 for details), and records of species occurrence and habitats where each species was recorded (Appendix S2) for each locality. We considered only sufficiently sampled localities with a near complete local checklist where samplings were carried out by expert ornithologists. After building a database, we selected only the species of the order Passeriformes and organized the data into an occurrence matrix with both Passeri and Tyranni assemblages. The data were standardized by removing migratory and aquatic species, as their distributions may not be affected by the processes evaluated in this study, which would increase the residuals of the analyses. We included both the species occurring in one habitat type (forest or savanna) and the species that are more flexible, that is, those occurring in both habitats. We also considered the nomenclature updates and corrections of species records from the inventories evaluated by Lees et al. (2015). We evaluated the spatial autocorrelation of assemblages using Mantel's statistic (permutation = 999), performed in ade4 R package (Dray & Dufour, 2007), and there was no significant spatial autocorrelation (*r* = .047, *p* = .056).

### 2.2 Ecological traits

We obtained information on 18 functional ecological traits for each species from Wilman et al. (2014), a database that has been used in studies on the functional diversity of birds (e.g., Barbet‐Massim & Jetz, 2015; Schipper et al., 2016; Sobral, Lees, & Cianciaruso, 2016). These traits have been widely used because they provide information on how species interact with each other, how they use the resources within their habitats of occurrence, and what functions they have in the ecosystem. We used the following traits: diet, treated as the estimated proportion of use of each diet item (1—invertebrates; 2—mammals, birds; 3—reptiles, snakes, amphibians, salamanders; 4—fish; 5—vertebrates general or unknown, for species where it was not clear what kind of vertebrates were being eaten; 6—scavenge, garbage, offal, carcasses, trawlers, carrion; 7—fruit, drupes; 8—nectar, pollen, plant exudates, gums; 9—seeds, maize, nuts, spores, wheat, grains; 10—other plant material), foraging stratum, treated as the estimated percentage of time spent in each strata (11—water, foraging on or just below (<5 inches) water surface; 13—ground, 14—understory, 15—mid to high levels, 16—canopy, 17—aerial), and 18—body mass as a continuous variable. The diet and forage stratum were based on the estimated proportion of use of each food item and of each stratum (“fuzzy” variables *sensu* Pavoine, Vallet, Dufour, Gachet, & Daniel, 2009), respectively, wherein items sum 100% total for each species. For example, a species can have a diet composed of 60% invertebrates and 40% endothermic vertebrates (see Wilman et al., 2014 for more details). For the 21 species (4.43%) absent from this database, we repeated the ecological traits of phylogenetically close species. These missing species include newly elevated subspecies at the species level (*splits*), or a new species described for science (see Appendix S3 for details). This information was organized into a matrix of species versus ecological traits containing both Passeri and Tyranni species.

### 2.3 Phylogenetic tree

To quantify phylogenetic diversity we used the proposed BirdTree (http://www.birdtree.org), a dated global phylogeny that contains about 10,000 bird species (Jetz, Thomas, Joy, Hartmann, & Mooers, 2012) based largely on molecular data (e.g., Kennedy et al., 2014; Sobral et al., 2016). This phylogeny includes almost all species sampled in the present study (95.57%). To reduce the potential effect of phylogenetic uncertainties, we built a phylogeny of maximum credibility value (MCC, maximum clade credibility), from 9,999 random, complete, and dated phylogenies (the same used in Sobral et al., 2016). For this, we used the TreeAnnotator v1.8.1 software, part of the BEAST v1.8.1 package (Drummond, Suchard, Xie, & Rambaut, 2012). A total of 4.43% of the species in our study were absent from the phylogeny of Jetz et al. (2012) as they constitute *splits* or new descriptions for science and were therefore inserted into the MCC tree as polytomies of close species (see Appendix S3 for details). Subsequently, we extracted the phylogenetic relationships only for the species used in the study (145 Passeri species and 329 Tyranni species).

### 2.4 Functional and phylogenetic alpha diversity

To calculate the functional diversity of assemblages, we converted the ecological traits matrix into a similarity matrix using a modified Gower distance (Pavoine et al., 2009). This measure quantifies the functional distance between all species by assigning equal weights to different types of variables (proportions and continuous variables in our study) (Pavoine et al., 2009). Then, using the distance matrix among all species, we quantified the functional diversity of the Tyranni and Passeri assemblages through Rao's quadratic entropy (Botta‐Dukát, 2005; Rao, 1982), an index of functional diversity that represents the average dissimilarity between all co‐occurring species in the same assemblage (Botta‐Dukát, 2005; Laliberté & Legendre, 2010). The Rao index is correlated to the number of species, but this correlation is especially strong at low species richness (which is not the case in our study), when the size of the dissimilarity matrix is small. This index will be greater when there are a greater number of functionally unique species (De Bello, Carmona, Lepš, Szava‐Kovats, & Pärte, 2016). We calculated Rao in the R environment using the “melodic” function, which computes Rao using both abundance data (when available) and the presence/absence data (as in our study) (De Bello et al., 2016). Therefore, we consider Rao's Entropy a good measure of the total functional diversity of our assemblages.

To determine phylogenetic diversity, we calculated the mean pairwise distance (MPD) and mean nearest taxon distance (MNTD). These measures are complementary and independent of species richness. The MPD consists of the mean phylogenetic distance between all pairs of species of the same assemblage and is considered a basal measure of the phylogenetic relationships of co‐occurring species as it captures the largest branches of the phylogenetic tree. The MNTD, in turn, quantifies the mean phylogenetic distance of each species to its nearest neighbor in the phylogenetic tree with which they co‐occur in the assemblage and is considered a terminal measure of the phylogenetic relationships of co‐occurring species (Webb, 2000). Thus, MPD potentially captures the relationships between older species, while MNTD reveals patterns about the relationships among the most recent species. We calculated both indices using the “picante” package (Kembel et al., 2010) of the R environment using the functions “mpd” and “mntd,” respectively.

We calculated the species richness as proportions of the total number of Passeriformes species in each habitat type. We compared species richness, functional diversity, and phylogenetic diversity between Passeri and Tyranni in each type of habitat using paired *t*‐tests when data showed normal distribution (according to Shapiro–Wilk test) and the Mann–Whitney *U*‐test when data did not met the assumption of normality.

### 2.5 Functional beta diversity

To test the hypothesis that there must be greater turnover in ecological traits between Passeri and Tyranni in forests than in savannas, we calculated functional beta diversity through the UniFrac index. This index represents a measure of dissimilarity derived from the Jaccard similarity index, which allows us to decompose beta diversity into its turnover and nestedness components using a functional dendrogram (Leprieur et al., 2012; Lozupone, Hamady, & Knight, 2006). For the calculation, we used a functional dendrogram that contained both Passeri and Tyranni assemblages. UniFrac varies from 0 to 1, 0 indicating that the compared assemblages have identical functional composition, and 1 that the assemblages are completely different, that is, they do not share any branch of the functional dendrogram (Leprieur et al., 2012). We performed the analyses in the R environment using the functions available in Leprieur et al. (2012). We calculated the UniFrac between all pairs of assemblages and subsequently extracted the mean functional beta diversity generated between pairs of Passeri and Tyranni assemblages in each habitat type.

To investigate the ecological traits of Passeri and Tyranni species most associated with each habitat type, we performed a principal component analysis (PCA) using the “FactoMineR” package in the R environment. We used the same ecological traits considered in functional alpha diversity analysis, that is, diet and forage stratum (percentage), and body mass (continuous). Thus, we calculated two PCA, one for Passeri assemblages and another for Tyranni.

## 3 RESULTS

### 3.1 Functional and phylogenetic alpha diversity

**Figure 2:**
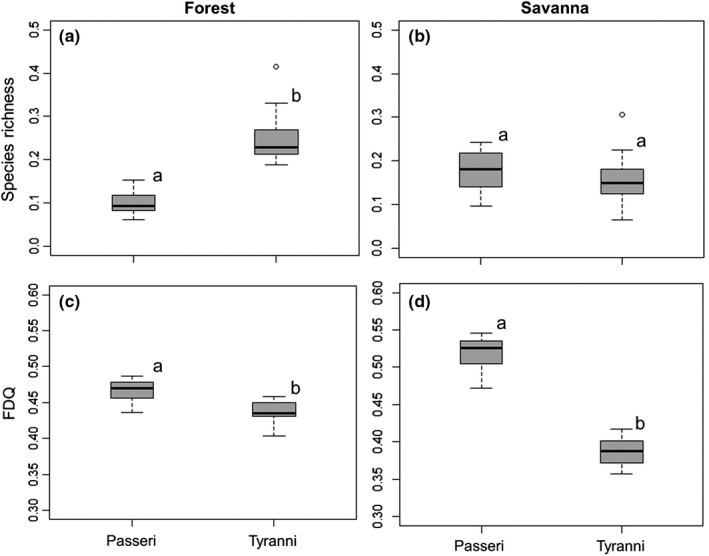
Proportional species richness (a and b) and functional diversity (FD_*Q*_) values (c and d) for the assemblages of Passeri and Tyranni in forests and savannas of the Brazilian Amazon. Pairs with different letters differed statistically (*p* < .05) when compared through a paired *t*‐test or Mann–Whitney *U*‐test

We recorded 474 Passeriformes species from the compiled data (145 Passeri and 329 Tyranni). A total of 350 species occurred exclusively in forests (80 of which were Passeri and 270 Tyranni), while 65 species were recorded only in savanna areas (39 Passeri and 26 Tyranni). Fifty‐nine species occurred in both habitats, 26 Passeri and 33 Tyranni. In forest areas, the relative richness of Tyranni was higher (mean = 0.24; *SD* = 0.05) when compared to Passeri species richness (mean = 0.09, *SD* = 0.02) (*t* = −16.92, *df* = 21, *p* < .001, Figure 2a). However, the relative richness of Passeri (mean = 0.17; DP = 0.05) and Tyranni (mean = 0.15; DP = 0.06) did not differ in savanna areas (*t* = 1.09; *df* = 11; *p* = .30) (Figure 2b).

Contrary to our expectations, in forest areas the functional diversity of Passeri (mean = 0.46, *SD* = 0.01) was greater than the functional diversity of Tyranni (mean = 0.43, *SD* = 0.01) (Mann–Whitney *U* = 252; *p* < .001; Figure 2c). Similarly, in savanna habitat, Passeri assemblages showed higher functional diversity (mean = 0.51, *SD* = 0.03) than Tyranni (mean = 0.38, *SD* = 0.06) (Mann–Whitney *U* = 78; *p* < .001; Figure 2d), supporting our hypothesis.

**Figure 3:**
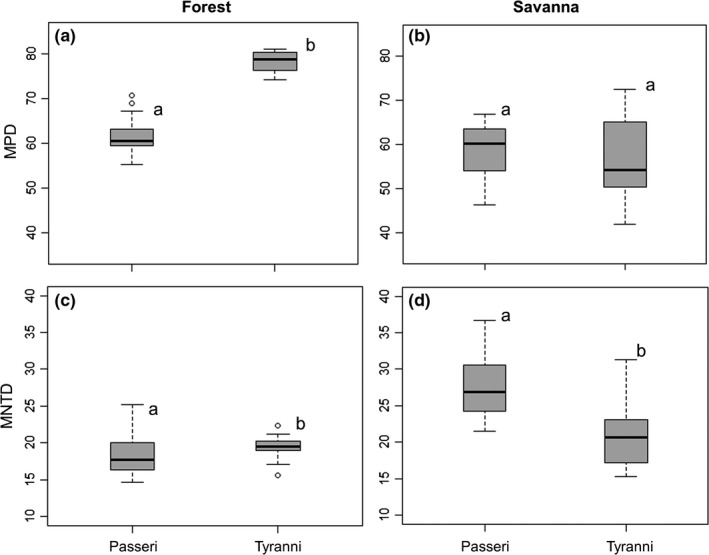
Mean pairwise distance (MPD) (a and b) and mean nearest taxon distance (MNTD) (c and d) recorded for the Passeri and Tyranni assemblages in forests and savannas of the Brazilian Amazon. Pairs indicated with distinct letters differed statistically (*p* < .05) when compared through a paired *t*‐test or Mann–Whitney *U*‐test

In forest areas, Tyranni presented a higher mean phylogenetic distance (mean = 78.51, *SD* = 1.80) than Passeri (mean = 62.08, *SD* = 3.01) (*t* = −21.19, *df* = 21, *p* < .001, Figure 3a). However, in savanna areas, the two suborders presented similar mean phylogenetic distances (Passeri: mean = 58.48, *SD* = 6.16; Tyranni: mean = 56.54, *SD* = 10.10) (Mann–Whitney *U* = 44, *p* = .73, Figure 3b). In forest areas, Passeri presented higher mean phylogenetic distances between nearest neighbors (mean = 18.93, *SD* = 1.37) than Passeri (mean = 17.17, *SD* = 1.70) (Mann–Whitney *U* = 41, *p* < .004, Figure 3c). In savanna, the mean phylogenetic distance between nearest neighbors was higher for Passeri (mean = 27.59, *SD* = 4.48) than for Tyranni (mean = 20.81, *SD* = 4.50) (Mann–Whitney *U* = 77; *p* < .001; Figure 3d).

### 3.2 Functional beta diversity

**Figure 4:**
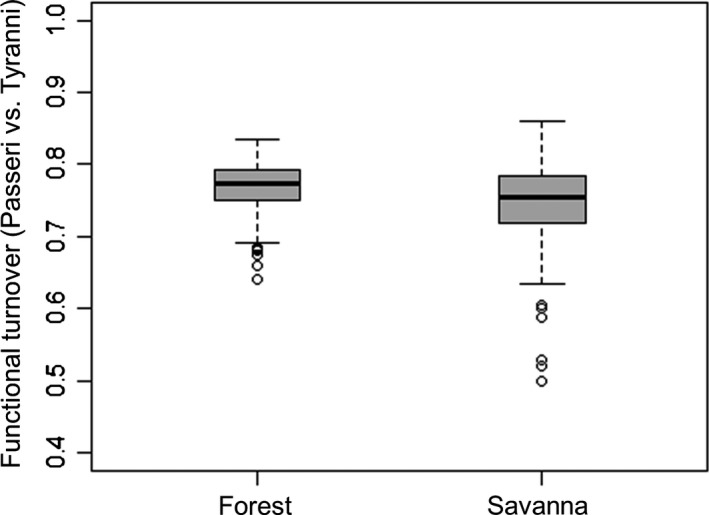
Boxplots representing median, 25% and 75% quartiles and maximum and minimum values of functional turnover between Passeri and Tyranni assemblages in forests and savannas of the Brazilian Amazon

The functional beta diversity between pairs of Passeri and Tyranni assemblages was high (~80%) for both forest and savanna habitats, and there was greater contribution of the turnover (~75%) than of the nestedness component (~5%) in both habitats (Table 1). In addition, the functional turnover values between Passeri and Tyranni assemblages in forests and savannas were similar, failing to support our assumption that forest areas should exhibit greater functional turnover between Passeri and Tyranni assemblages than savanna areas (Table 1, Figure 4).

**Table 1:** Mean values of functional beta diversity between pairs of assemblages of Passeri and Tyranni occurring in forests and savannas of the Brazilian Amazon. The standard deviation of the beta diversity components is presented in parentheses, next to their respective mean values

Habitat	Functional beta diversity	*Turnover*	Nestedness
Forest	0.80 (0.01)	0.77 (0.03)	0.03 (0.02)
Savanna	0.80 (0.03)	0.74 (0.06)	0.06 (0.05)

**Figure 5:**
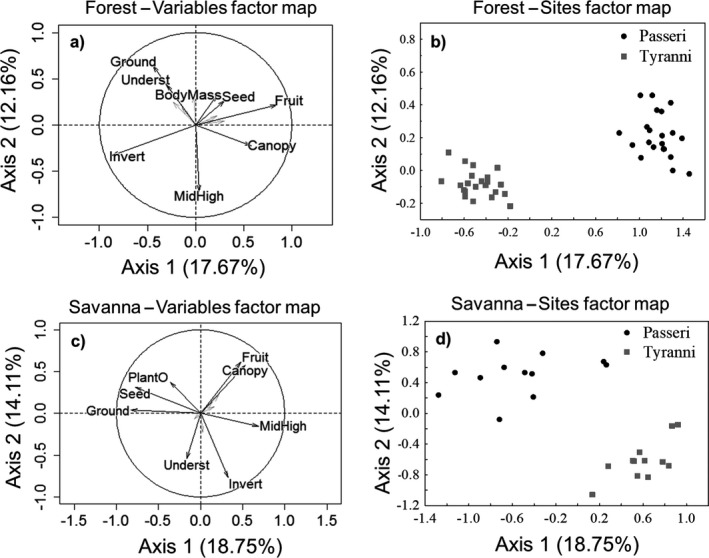
Principal components analysis (PCA) highlighting the distribution of the ecological traits (a and c) and the assemblages (b and d) of Passeri and Tyranni in forests and savannas of the Brazilian Amazon. Foraging stratum: ground, Underst = understory, MidHigh = mid to high levels, canopy. Diet: Invert = invertebrates, Fruit, Seed, PlantO = diverse plant material; and body mass

The first two PCA axes explained 29.83% of the variation in the ecological traits of the Passeri and Tyranni assemblages in forest areas. The traits most related to Passeri were feeding on fruits and seeds, and foraging in the canopy, while Tyranni was more represented by species that feed on invertebrates and forage in the ground and understory (Figure 5a–b). In savannas, the two axes explained 32.86% of the variation, where the most representative ecological traits of Passeri species were feeding on fruits, seeds, and other diverse plant materials, and foraging in the ground and canopy, while Tyranni was more represented by species that forage in the low, medium, and high vegetation strata, and that feed on invertebrates (Figure 5c–d).

## 4 DISCUSSION

### 4.1 Functional and phylogenetic alpha diversity

Some studies have discussed the distribution and diversification of Passeriformes in the New World (Ericson et al., 2014; Jønsson et al., 2015; Kennedy et al., 2014; Ohlson, Irestedt, Ericson, & Fjeldså, 2013); however, in our study we applied measures of functional and phylogenetic diversity in order to discuss the patterns of diversity of Passeri and Tyranni in forest and savanna habitats in the Amazon, considering the biogeography of these suborders and their ecological characteristics.

Tyranni species richness was greater than Passeri species richness in forest areas; however, in savanna areas, there was no difference between the number of species of the two suborders. A number of bird inventories in Amazonia have pointed to a greater richness of Tyranni assemblages in forest areas (Schunck et al., 2011), and a greater diversity of Passeri in open vegetation (Sanaiotti & Cintra, 2001); however, this pattern had not yet been statistically tested at the time of writing. Ricklefs (2002) observed that in South America Tyranni present greater richness in the forest interior, while Passeri are prominent in open habitats. In our study, we did not observe differences between the two suborders in savanna environments as the Tyrannidae family (Tyranni suborder) shares habitat preferences with the Passeri, also occupying mainly open habitats (Kennedy et al., 2014; Ohlson, Fjeldså, & Ericson, 2008).

Contrary to our hypothesis, although Tyranni are dominant in forest, Passeri presented greater functional diversity, considering the set of traits used in this study. Passeri exhibit a surprising diversity of biological and behavioral characteristics (Barker et al., 2004), making them one of the most successful bird groups in occupying different habitat types (Feduccia, 1999), which may also have contributed to the functional diversity patterns found for this group. In agreement with our results, some studies have shown that assemblages of forest birds with high species richness present a greater number of functionally similar species (functional redundancy) (Almeida et al., 2016; Prescott et al., 2016).

In savanna areas, the functional diversity of Passeri assemblages was also greater than that of Tyranni assemblages, supporting our hypothesis. Passeri species have a greater capacity for occupying open habitats, can explore a wider range of available resource types, and can use different strategies to obtain these resources (Feduccia, 1999; Newton, 2003; Ricklefs, 2002; Swanson & Bozinovic, 2011). These characteristics may have allowed a higher functional diversity for this group compared to Tyranni in savanna areas.

Our results show that Tyranni have a greater mean phylogenetic distance than Passeri in forests. This supports the theory that one of the main factors responsible for the diversification of this suborder in tropical forests was the ancient colonization of this type of habitat, and the long period of time available for the speciation and appearance of a great number of lineages (Vuilleumier, 1985; Wiens & Donoghue, 2004). Because of their low dispersal capacity, understory birds, like many Tyranni, have a greater genetic divergence when compared to canopy birds (Burney & Brumfield, 2009; Hawkins, Diniz‐Filho, Jaramillo, & Soeller, 2006) and this may also have led to a high diversity of lineages within this suborder (Smith et al., 2014). In addition, forests with greater structural complexity and productivity, such as those existing in South America, allow greater diversification and persistence of species, through having a higher number and variety of available niches (Dreiss et al., 2015).

The Tyranni and Passeri assemblages showed similar mean phylogenetic distances in savanna areas. This result may be related to the fact that more severe, less complex, and productive habitats such as savannas tend to restrict the persistence of fewer lineages, therefore presenting assemblages with lower phylogenetic diversity (Dreiss et al., 2015). Unlike the high diversification observed in forest areas, only a few Tyranni lineages are typical of open habitats, such as the Tyrannidae family (Kennedy et al., 2014). Although Passeri species have a greater capacity to occupy open habitats, the average genetic divergence covered by the basal nodes of this group in South America represents about a third of the diversification time for Tyranni (Ricklefs, 2002).

Our hypothesis that Passeri would present more recent lineages in forests when compared to Tyranni was not supported. We found that the mean nearest taxon distance of Tyranni was higher than Passeri in this habitat type, revealing that there is a higher diversity of more recent lineages for this suborder in forests. This result may be due to the fact that clades which originate in the tropics, such as Tyranni, presented higher diversification rates (Wiens, 2011; Wiens & Donoghue, 2004). In addition, because forests are highly productive environments, it allows both persistence of old lineages and *in situ* speciation, especially for groups that have primarily evolved in this type of habitat (Burney & Brumfield, 2009; Ricklefs, 2006; Wiens & Donoghue, 2004).

Passeri presented a greater mean nearest taxon distance than Tyranni in savanna areas. This can be explained by the fact that Passeri represent the highest known bird radiation (Barker et al., 2004; Kennedy et al., 2014) and that several lineages occupy savanna areas (e.g., Emberizidae, Icteridae, Thraupidae). In addition, the range expansion of Passeri and the colonization of new ecological space may have promoted rapid species diversification that results in more recent lineages, which show short internodes in the phylogenetic tree (Kennedy et al., 2014; Rabosky et al., 2014).

### 4.2 Functional beta diversity

Our results show a high functional turnover between Passeri and Tyranni assemblages in both forests and savannas. In general, we observed that in forest areas Passeri assemblages predominantly occupy the canopy and feed on fruits and seeds, similar to that found by Ricklefs (2002). Tyranni, in turn, dominate the forest interior and feed mainly on large insects (Ricklefs, 2002). In open areas, Passeri has many representatives that use seeds and fruits as their main food resources, while Tyranni is primarily represented by birds of the family Tyrannidae, with a diet composed mainly of small insects (Ricklefs, 2002). Our findings show that the two suborders occupy different niches and consume different resources, not only in structurally complex and highly productive habitats (i.e., forests), but also in habitats with less variety of resources (i.e., savannas).

Our results indicate that the phylogenetic and functional diversity patterns of Passeri and Tyranni assemblages in forests and savannas seem to reflect not only the different biogeographic histories shown by the two suborders (Kennedy et al., 2014), but also of the differences of occurrence within each habitat type (Ricklefs, 2002). In addition, the diversity patterns that we found suggest that the suborders have different ecological strategies that avoid a high niche overlap and, potentially, strong antagonistic interactions (Jønsson et al., 2015; Lovette & Hochachka, 2006).

## CONFLICT OF INTEREST

None declared.

## AUTHORS’ CONTRIBUTIONS

S.M.A. and M.P.D.S. conceived the initial idea of the work; S.M.A., L.J., and F.L.S. conducted the analyses; S.M.A led the writing and all authors contributed with the revision of the manuscript.
