# Supplementary material for: The influence of biogeographic history on the functional and phylogenetic diversity of passerine birds in savannas and forests of the Brazilian Amazon
Source: Ecol Evol. 2018 Mar 3;8(7):3617–27. doi: 10.1002/ece3.3904 (PMC5901182; doi:10.1002/ece3.3904)
Supplement: Supplementary file 1 [file ECE3-8-3617-s001.doc]

*Ecology and Evolution*

**Supporting Information**

### The influence of biogeographic history on the functional and phylogenetic diversity of Passerine birds in savannas and forests of the Brazilian Amazon

Sara M. Almeida, Leandro Juen, Fernando L. Sobral and Marcos P.D. Santos

**Appendix S1** Geographic coordinates and sampling method(s) of the bird inventories compiled in this study. Method: I) capture; II) visualization; III) vocalization; IV) literature; V) ornithological collection, and the richness of species in the suborders Passeri and Tyranni. (*) Information obtained from the database compiled by Santos (2005) and presented by Santos (2012).

**Appendix S2** Lists of Passeriformes species compiled of 22 forests areas (F) and 12 savannas areas (S) in the Brazilian Amazon. Sites (1 to 34) show where each species was recorded. Information on the sites are shown in Appendix S1. The nomenclature and taxonomic sequence follows the list of the Brazilian Committee of Ornithological Records (CBRO, Piacentini *et al*., 2015).

**Appendix S3** Birds species recorded in our studyand that are absent from the BirdTree phylogeny. These species were inserted as polytomies in the MCC tree used in this study. Species indicated with asterisks (*) constitute splits and those indicated with the number (¹) constitute new descriptions for science.

| **Appendix S1** Geographic coordinates and sampling method(s) of the bird inventories compiled in this study. Method: I) capture; II) visualization; III) vocalization; IV) literature; V) ornithological collection, and the richness of species in the suborders Passeri and Tyranni. (*) Information obtained from the database compiled by Santos (2005) and presented by Santos (2012). | | | | | | |
| --- | --- | --- | --- | --- | --- | --- |
| Ornithological localities | Coordinates | Sampling method |  | Passeri | Tyranni | References |
| **Florestas** |  |  |  |  |  |  |
| 1 - Roraima, Parque Nacional Viruá | 1°29’N, 61°00’W | I,II,III |  | 37 | 90 | Laranjeiras et al. 2011 |
| 2 - Amazonas, Pousada Rio Roosevelt | 08°29’S, 60°57’W | II, III |  | 42 | 123 | Whittaker 2009 |
| 3 - Maranhão, REBIO Gurupi | 3°50'S 46°42'W | I,II,III |  | 34 | 80 | Lima et al. 2014 |
| 4 - Mato Grosso, Reserva Florestal Cristalino | 9°41’S, 55°56’W | I,II,III |  | 58 | 135 | Lees et al. 2012 |
| 5 - Amazonas, Tefé, Urucu | 4°50'S, 65°16'W | I,II,III |  | 30 | 88 | Peres e Whittaker 1991; Wittaker et al. 2008 |
| 6 - Rondônia, Ji‑Paraná, Igarapé Lourdes | 10°26’S, 61°39'W | I,II,III |  | 29 | 89 | Santos et al. 2011 |
| 7 - Acre, RESEX do alto Juruá | 8° 46′S, 72°7′W | I,II,III |  | 63 | 170 | Whittaker et al. 2002, Guilherme 2012 |
| 8 - Amazonas, PARNA do Jaú | 2° 15’S, 62°38’W | I,II,III,V |  | 34 | 101 | Borges & Almeida 2011 |
| 9 - Amazonas, FLONA Pau-Rosa | 3° 44’S, 58° 17’W | I,II,III |  | 25 | 92 | Dantas et al. 2011 |
| 10 - Pará, FLONA do Tapajós | 3°21’S, 54°56’W | I,II,III,V |  | 48 | 110 | Henriques et al. 2003 |
| 11 - Amazonas, Reserva Adolph Ducke | 2°57’S, 59°55’W | I,II,III,V |  | 38 | 100 | Cohn-Haft et al. 1997 |
| 12 - Acre, Alto Rio Purus | 09°00’S, 69°32’W | I,II,III |  | 25 | 77 | Guilherme & Dantas 2011 |
| 13 - Acre, RESEX Chico Mendes | 10° 30’S, 69° 47’W | I,II,III |  | 39 | 123 | Mestre et al. 2010 |
| 14 - Pará, Tailândia, Complexo Agropalma | 02°36’S, 48°44’W | I,II,III |  | 47 | 86 | Portes et al. 2011 |
| 15 - Pará, Tomé-Açu | 02°30’S, 48°17’W | I,II,III |  | 40 | 80 | Portes et al. 2011 |
| 16 - Pará, Cauaxi | 03°04’S, 48°17’W | I,II,III |  | 34 | 89 | Portes et al. 2011 |
| 17 - Pará, Rio Capim | 03°04’S, 48°33’W | I,II,III |  | 36 | 95 | Portes et al. 2011 |
| 18 - Amapá, RESEX Rio Cajari | 00°34’S, 52°16’W | I,II,III |  | 38 | 109 | Schunck et al. 2011 |
| 19 - Roraima, Amajari, ESEC Maracá | 3°25’N, 61°40’W | V |  | 59 | 100 | FMNH - MZUSP - D. F. Stotz (1997)* |
| 20 - Roraima, Mucajaí, Colônia de Apiaú | 2°34’N, 61°18’W | V |  | 54 | 110 | MPEG – Stotz (1997).; Borges (1994)* |
| 21 - Roraima, Pacaraima, Fronteira BR-VE | 4°25’N, 61°08’W | V |  | 27 | 87 | FMNH; MZUSP – Stotz (1997)* |
| 22 - Roraima, Município de São João Baliza | 0°58’N, 59°48’W | V |  | 31 | 78 | INPA - Cohn-Haft & Naka* |
| **Savanas** |  |  |  |  |  |  |
| 23 - Amapá, CE Cerrado – Embrapa | 0°2’N, 51°2’W | II, III |  | 20 | 15 | Boss & Silva 2014 |
| 24 - Roraima, Cantá, Fazenda Santa Cecília | 2°48’N, 60°49’W | |  | 18 | 17 | FMNH; MZUSP – Stotz (1997)* |
| 25 - Roraima, município de Boa Vista | 2°49’N, 60°40’W | |  | 30 | 21 | MPEG; MZUSP; FMNH - Stotz (1997)* |
| 26 - Roraima, Bonfim, Forte de São Joaquim | 3°01’N, 60°28’W | V |  | 12 | 14 | FMNH - MZUSP - D. F. Stotz (1997)* |
| 27 - Roraima, município de Mucajaí | 2°53’N, 61°42’W | |  | 27 | 28 | Pinto (1966)* |
| 28 - Roraima, Pacaraima, Vila Sorocaima | 4°22’N, 61°00’W | |  | 54 | 17 | FMNH - MZUSP - D. F. Stotz (1997)* |
| 29 - Sipaliwini, Fronteira Brazil-Suriname | 1°57’N, 55°55’W | I, II, III |  | 13 | 8 | Mittermeier et al. 2010 |
| 30 - Amapá, Vilanova | 0°09’N, 51°32’W | I,II,III |  | 25 | 16 | Schunck et al. 2011 |
| 31 - Pará, Alter-do-Chão | 2°31’S, 55°00’W | I, II, III |  | 19 | 20 | Sanaiotti & Cintra 2001 |
| 32 - Pará, município de Monte Alegre | 1°56’S, 54° 03’W | I, II, III, V | | 22 | 24 | Vasconcelos et al. 2011 |
| 33 - Amazonas, município de Manicoré | 8°33’S, 61°26’W | I, II, III |  | 17 | 20 | Aleixo & Poletto 2007 |
| 34 - Pará, Serra do Cachimbo | 09°22’S, 54°54’W | I, II, III |  | 25 | 38 | Santos et al. 2011 |

**References**

Aleixo, A. & Poletto, F. (2007) Birds of an open vegetation enclave in Southern Brazilian Amazonia. *Wilson Journal of Ornithology*, **119**, 610–630.

Borges, S.H. & Almeida, R.A. (2011) Birds of the Jaú National Park and adjacent area, Brazilian Amazon: new species records with reanalysis of a previous checklist. *Revista Brasileira de Ornitologia*, **19**, 108–133.

Boss, R.L. & Silva, J.M.C. (2014) Core and transient species in an Amazonian savanna bird assemblage. *Revista Brasileira de Ornitologia*, **22**, 374–382.

Cohn-Haft, M., Whittaker, A. & Stouffer, P.C. (1997) A New Look at the “Species-Poor” Central Amazon: The Avifauna North of Manaus, Brazil. *Ornithological* *Monographs*, **48**, 205–235.

Dantas, S., Faccio, M.S. & Lima, M.F. (2011) Avifaunal inventory of the Floresta Nacional de Pau-Rosa, Maués, State of Amazonas, Brazil. *Revista Brasileira de Ornitologia*, **19**, 154–166.

Guilherme, E. & Dantas, S. M. 2011. Avifauna of the upper Purus river, State of Acre, Brazil. *Revista Brasileira de Ornitologia*, **19**, 185–199.

Guilherme, E. (2012) Birds of the Brazilian state of acre: diversity, zoogeography, and conservation. *Revista Brasileira de Ornitologia*, **20**, 393–442.

Henriques, L.M.P., Wunderle, J.M. & Willig, M.R. (2003) Birds of the Tapajós National Forest, Brazilian Amazon: a preliminary assessment. *Ornitologia* *Neotropical*, **14**, 307–338.

Laranjeiras, T.O., Naka, L.N., Bechtoldt, C.L., Costa, T.V.V., Andretti, C.B., Cerqueira, M.C., Torres, M.F., Rodrigues, G.L., Santos, M.P.D., Vargas, C.F., Pacheco, A.M.F., Sardelli, C.H., Mazar Barnett, J. & Cohn-Haft, M. (2014). The avifauna of Viruá National Park, Roraima: megadiversity in northern Amazonia. *Revista Brasileira de Ornitologia*, **2**, 138–171.

Lees, A. C., Zimmer, K. J., Marantz, C.A., Whittaker, A., Davis, B.J.W. & Whitney, B.M. (2013) Alta Floresta revisited: an updated review of the avifauna of the most intensively surveyed locality in south-central Amazonia. *Bulletin of the British Ornithologists’ Club*, **133**, 178–239.

Lima, D.M., Martínez, C. & Raíces, D.S.L. (2014) An avifaunal inventory and conservation prospects for the Gurupi Biological reserve, Maranhão, Brazil. *Revista Brasileira de Ornitologia*, **22**, 317–340.

Mestre, L.A.M., Thom, G., Cochrane, M.A. & Barlow, J. (2010) The birds of Reserva Extrativista Chico Mendes, South Acre, Brazil. Boletim do Museu Paraense Emílio Goeldi. *Ciências Naturais*, **5**, 311–333.

Mittermeier, J.C., Zyskowski, K., Stowe, E.S. & Lai, J.E. (2010) Avifauna of the Sipaliwini Savanna (Suriname) with Insights into Its Biogeographic Affinities. *Bulletin of the Peabody Museum of Natural History*, **51**, 97–122.

Peres, C. & Whittaker, A. (1991) Annotated checklist of the bird species of the upper Rio Urucu, Amazonas, Brazil. *Bulletin of the British Ornithologists’ Club*, **111**, 156–171.

Pinto, O. M. O. *1966. Estudo crítico e catálogo remissivo das aves do Território Federal de Roraima.* Cadernos da Amazônia, Manaus, **8**, 1–176.

Portes, C.E.B., Carneiro, L.S., Schunck, F., Silva, M.S., Zimmer, K.J., Whittaker, A., Poletto, F., Silveira, L.F. & Aleixo, A. (2011) Annotated checklist of birds recorded between 1998 and 2009 at nine areas in the Belém area of endemism, with notes on some range extensions and the conservation status of endangered species. *Revista Brasileira Ornitologia*, **19**,167–184.

Sanaiotti, T.M. & Cintra. (2001) Breeding and Migrating Birds in an Amazonian Savanna. *Studies on Neotropical Fauna and Environment*, **36*,*** 23–32.

Santos, M.P.D. (2005) *Avifauna do estado de Roraima: Biogeografia e Conservação*. PhD Thesis, Universidade Federal do Pará, Belém, 589p.

Santos, M.P.D., Silveira, L.F. & Silva, J.M.C. (2011) Birds of Serra do Cachimbo, Pará State, Brazil. *Revista Brasileira de Ornitologia*, **19**, 244–259.

Santos, M.P.D. (2012) A gap analysis of ornithological research in the Brazilian state of Roraima. *Biota Neotropica*, **12,** http://www.biotaneotropica.org.br/v12n2/en/abstract?article+bn01312022012 ISSN 1676-0603.

Schunck, F., De Luca, A.C, Piacentini, V.Q., Rego, M.A., Rennó, B. & Corrêa, A.H. (2011) Avifauna of two localities in the south of Amapá, Brazil, with comments on the distribution and taxonomy of some species. *Revista Brasileira de Ornitologia*, **19**, 93–107.

Stotz, D.F., Lanyon, S.M., Schulenberg, T.S., Willard, D.E., Peterson, A.T. & Fitzpatrick, J.W. (1997) An avifaunal survey of two tropical forest localities on the middle rio Ji‑Paraná, Rondônia, Brazil. *Ornithological Monographs*, **48**, 763–781.

Vasconcelos, M.F, Dantas, S.M. & Silva, J.M.C. (2011) Avifaunal inventory of the Amazonian savannas and adjacent habitats of the Monte Alegre region (Pará, Brazil), with comments on biogeography and conservation. *Boletim do Museu Paraense Emílio Goeldi. Ciências Naturais*, **6**, 119–145.

Whittaker, A. (2009) Pousada Rio Roosevelt: a provisional avifaunal inventory in south-western Amazonian Brazil, with information on life history, new distributional data and comments on taxonomy. *Cotinga*, **31**, OL 20–43.

Whittaker, A., Oren, D.C., Pacheco, J.F., Parrini, R. & Minns, J.C. (2002) Aves registradas na Reserva Extrativista do Alto Juruá. Enciclopédia da floresta: O Alto Juruá: Práticas e Conhecimentos das Populações (ed. by Carneiro da Cunha, M. & Almeida, M.B.), pp. 81–99. Companhia das Letras, São Paulo.

**Appendix S2** Lists of Passeriformes species compiled of 22 forests areas (F) and 12 savannas areas (S) in the Brazilian Amazon. Sites (1 to 51) show where each species was recorded. Information on the sites are shown in Appendix S1. The nomenclature and taxonomic sequence follows the list of the Brazilian Committee of Ornithological Records (CBRO, Piacentini et al., 2015).

| **Taxon** | **Localities** | **Habitat** |
| --- | --- | --- |
| **Passeriformes** |  |  |
| **Tyranni** |  |  |
| **Thamnophilidae** |  |  |
| *Euchrepomis humeralis* | 2,5,7,20 | F |
| *Euchrepomis spodioptila* | 8,11,18,21 | F |
| *Myrmornis torquata* | 4,9,11,16-18,20 | F |
| *Pygiptila stellaris* | 1,2,3,4,6,7,8,9,12,19,21 | F |
| *Microrhopias quixensis* | 1,2,4,6,7,9,13,18,22 | F |
| *Neoctantes niger* | 7,13 | F |
| *Clytoctantes atrogularis* | 2 | F |
| *Epinecrophylla gutturalis* | 11,18 | F |
| *Epinecrophylla leucophthalma* | 2,4,6,12,13 | F |
| *Epinecrophylla haematonota* | 2,5-7,8 | F |
| *Epinecrophylla pyrrhonota* | 19,20,21 | F |
| *Epinecrophylla ornata* | 2,4,12,13 | F |
| *Myrmophylax atrothorax* | 1,6,7,11,12,19-21,22 | F |
| *Myrmotherula brachyura* | 1,2,4-6,7,8,9,11,13,18-20,21,22 | F |
| *Myrmotherula obscura* | 7 | F |
| *Myrmotherula ambigua* | 8,16 | F |
| *Myrmotherula sclateri* | 2,4,7,9,13 | F |
| *Myrmotherula surinamensis* | 22 | F |
| *Myrmotherula multostriata* | 2,3,7 | F |
| *Myrmotherula axillaris* | 1,2,3,4,5-10,11,12-20,22 | F |
| *Myrmotherula longipennis* | 1-4,5-17,20,21,22 | F |
| *Myrmotherula iheringi* | 2,7,9 | F |
| *Myrmotherula menetriesii* | 2-4,5,7-8,9,11,13-18,19,20,21 | F |
| *Formicivora grisea* | 24-27,30-34 | S |
| *Formicivora rufa* | 23,30-34 | S |
| *Isleria hauxwelli* | 2,3,4,5,6,9,12,13 | F |
| *Isleria guttata* | 1,11,18-20,21 | F |
| *Thamnomanes ardesiacus* | 7,8,11,12,13,18,19,20,21 | F |
| *Thamnomanes saturninus* | 2,4,5,6,7,9 | F |
| *Thamnomanes caesius* | 1-4,5,6,8-11,14-18,20,21,22 | F |
| *Thamnomanes schistogynus* | 7,12,13 | F |
| *Dichrozona cincta* | 2,4,6,7,8,9 | F |
| *Megastictus margaritatus* | 2,5,8,9 | F |
| *Dysithamnus mentalis* | 3,14,16,17 | F |
| *Herpsilochmus sellowi* | 34 | S |
| *Herpsilochmus stotzi* | 2 | F |
| *Herpsilochmus sticturus* | 18 | F |
| *Herpsilochmus stictocephalus* | 18 | F |
| *Herpsilochmus dorsimaculatus* | 1,8,11,20,21,22 | F |
| *Herpsilochmus rufimarginatus* | 1,2-4,6,14-17,19,20 | F |
| *Thamnophilus doliatus* | 2,6,13,19,20,23,26,27,33,34 | F,S |
| *Thamnophilus torquatus* | 34 | S |
| *Thamnophilus palliatus* | 4,14,15 | F |
| *Thamnophilus schistaceus* | 2,4,5,6,7,8,9,10,12,13,15,17 | F |
| *Thamnophilus murinus* | 1,5,7,8,11,18-20,21,22 | F |
| *Thamnophilus punctatus* | 1,18-21,22 | F |
| *Thamnophilus stictocephalus* | 6,9,15,33,34 | F,S |
| *Thamnophilus aethiops* | 2,3,4,5-7,8-10,12-17,19,20,21,22 | F |
| *Thamnophilus amazonicus* | 1,2-4,6,7,14-18 | F |
| *Cymbilaimus lineatus* | 1,2,4,6-8,9,11-13,18,20,22 | F |
| *Cymbilaimus sanctaemariae* | 7,12 | F |
| *Taraba major* | 1,7,13,14,19,20,21,34 | F,S |
| *Frederickena viridis* | 1,11,18,20 | F |
| *Frederickena unduliger* | 7.8 | F |
| *Myrmoderus ferrugineus* | 1,2,11,18 | F |
| *Hypocnemoides maculicauda* | 7,9,14,15 | F |
| *Hylophylax naevius* | 1,2,4,5-7,8,9-13,18,20,21,22 | F |
| *Sclateria naevia* | 5,6,7,9,10,14,15,22 | F |
| *Myrmelastes schistaceus* | 7 | F |
| *Myrmelastes hyperythrus* | 5,7,13 | F |
| *Myrmelastes rufifacies* | 4,6,9 | F |
| *Myrmelastes leucostigma* | 1,10,11,18,22 | F |
| *Myrmelastes humaythae* | 5,7,13 | F |
| *Myrmeciza longipes* | 18-20 | F |
| *Myrmoborus lophotes* | 7,13 | F |
| *Myrmoborus myotherinus* | 2,4,5-7,8,9,10,12,13,20,21 | F |
| *Myrmoborus leucophrys* | 1,4,6,7,12,13,19,21,22 | F |
| *Pyriglena leuconota* | 3,4,14-17 | F |
| *Percnostola rufifrons* | 11,18,22 | F |
| *Percnostola subcristata* | 1 | F |
| *Percnostola minor* | 8 | F |
| *Akletos goeldii* | 7,12,13 | F |
| *Hafferia fortis* | 5,7,12,13 | F |
| *Sciaphylax hemimelaena* | 2,4,7,12,13 | F |
| *Cercomacra manu* | 4,7,12 | F |
| *Cercomacra cinerascens* | 4,5,6,7,8,9-18,19,20,21,22 | F |
| *Cercomacroides nigrescens* | 4,6,7,9,12 | F |
| *Cercomacroides laeta* | 1,3,14-17,22 | F |
| *Cercomacroides tyrannina* | 1,11,18,19-21,22 | F |
| *Cercomacroides serva* | 7,13 | F |
| *Drymophila devillei* | 4,7,13 | F |
| *Hypocnemis hypoxantha* | 4,7,8 | F |
| *Hypocnemis subflava* | 6,13 | F |
| *Hypocnemis cantator* | 1,11,18-20,21,22 | F |
| *Hypocnemis peruviana* | 5,7,12,13 | F |
| *Hypocnemis striata* | 2,4,9,10 | F |
| *Pithys albifrons* | 1,11,18,19,20,21,22 | F |
| *Willisornis poecilinotus* | 2,5-9,11,12,13,18-20,21 | F |
| *Willisornis vidua* | 3,4,14-17 | F |
| *Phlegopsis nigromaculata* | 2-4,6,7,9,10,12-17 | F |
| *Phlegopsis borbae* | 2,9 | F |
| *Phlegopsis erythroptera* | 5,7,8 | F |
| *Gymnopithys leucaspis* | 8 | F |
| *Gymnopithys rufigula* | 1,11,18,19-21,22 | F |
| *Oneillornis salvini* | 5,7,12,13 | F |
| *Rhegmatorhina gymnops* | 4 | F |
| *Rhegmatorhina berlepschi* | 9 | F |
| *Rhegmatorhina hoffmannsi* | 2,6 | F |
| *Rhegmatorhina cristata* | 8 | F |
| *Rhegmatorhina melanosticta* | 5,7,13 | F |
| **Melanopareiidae** |  |  |
| *Melanopareia torquata* | 33,34 | S |
| **Conopophagidae** |  |  |
| *Conopophaga aurita* | 2,4,5,6,8,11,22 | F |
| *Conopophaga roberti* | 14-17 | F |
| *Conopophaga melanogaster* | 2 | F |
| *Conopophaga peruviana* | 7,13 | F |
| **Grallariidae** |  |  |
| *Grallaria varia* | 2,4,6,8,9-11,16-18 | F |
| *Grallaria eludens* | 7 | F |
| *Hylopezus macularius* | 4,8,11,16-18 | F |
| *Hylopezus paraensis* | 3 | F |
| *Hylopezus berlepschi* | 4,9,12 | F |
| *Myrmothera campanisona* | 2,3,6,7,8,9-11,13,18,19,20 | F |
| **Rhinocryptidae** |  |  |
| *Liosceles thoracicus* | 2,6,7,9 | F |
| **Formicariidae** |  |  |
| *Formicarius colma* | 2,4-8,9-18,20-22 | F |
| *Formicarius analis* | 2-8,9-18,19,22 | F |
| *Formicarius rufifrons* | 7 | F |
| *Chamaeza nobilis* | 4,5 | F |
| **Scleruridae** |  |  |
| *Sclerurus macconnelli* | 2-6,7,11,12-17,20,22 | F |
| *Sclerurus rufigularis* | 2,3,4,6,3-8,9,11,16,18,20,21,29 | F |
| *Sclerurus caudacutus* | 2-8,9,11-13,16,18-20,22 | F |
| *Sclerurus albigularis* | 4,7 | F |
| **Dendrocolaptidae** |  |  |
| *Dendrocincla fuliginosa* | 1,2-6,7-9,11-18,20-22 | F |
| *Dendrocincla merula* | 1-6,7,8,9,11-16,19-21,22 | F |
| *Deconychura longicauda* | 1,2-9,11-14,16-18,19,20,21 | F |
| *Sittasomus griseicapillus* | 2,4-8,9,11-13,18-20,22 | F |
| *Certhiasomus stictolaemus* | 2,4,5,7,8,8,9,11,14-18 | F |
| *Glyphorynchus spirurus* | 1 to 22 | F |
| *Xiphorhynchus pardalotus* | 1,11,18-21,22 | F |
| *Xiphorhynchus chunchotambo* | 12,13 | F |
| *Xiphorhynchus ocellatus* | 5,8,9 | F |
| *Xiphorhynchus elegans* | 2,4,6-7,12,13 | F |
| *Xiphorhynchus spixii* | 4,14-17 | F |
| *Xiphorhynchus obsoletus* | 1,6,14,15,18-20 | F |
| *Xiphorhynchus guttatus* | 3-8,9,10,12-20,22 | F |
| *Campylorhamphus trochilirostris* | 7,13 | F |
| *Campylorhamphus probatus* | 2,6,9 | F |
| *Campylorhamphus procurvoides* | 4,5,11,21,22 | F |
| *Dendroplex picus* | 3,5,6,7,12,14,15,18-20,24-28,30,32 | F,S |
| *Lepidocolaptes angustirostris* | 23,29,31-33 | S |
| *Lepidocolaptes albolineatus* | 2,4,5,7,9,11,12,15-17,19,20 | F |
| *Lepidocolaptes duidae* | 8 | F |
| *Lepidocolaptes layardi* | 3 | F |
| *Dendrexetastes rufigula* | 1,2,3,6,7,10 | F |
| *Dendrocolaptes certhia* | 1,2,4,5,7,8,9-12,13,18-21,22 | F |
| *Dendrocolaptes medius* | 3,14-17 | F |
| *Dendrocolaptes picumnus* | 1,4,5,7-8,9,11,12,13,19,21 | F |
| *Dendrocolaptes hoffmannsi* | 2 | F |
| *Xiphocolaptes promeropirhynchus* | 2,4,5,7,8,9,10,13,19 | F |
| *Hylexetastes stresemanni* | 7,8,13 | F |
| *Hylexetastes perrotii* | 4,6,11,18,19 | F |
| *Hylexetastes uniformis* | 2,6,9,10 | F |
| **Xenopidae** |  |  |
| *Xenops tenuirostris* | 2,5,7,13 | F |
| *Xenops minutus* | 1-7,8,9-22 | F |
| *Xenops rutilans* | 4,7,14 | F |
| **Furnariidae** |  |  |
| *Berlepschia rikeri* | 25 | S |
| *Microxenops milleri* | 18 | F |
| *Furnarius leucopus* | 7,19,20,22,26,27 | F,S |
| *Ancistrops strigilatus* | 2,4,5,8 | F |
| *Clibanornis obscurus* | 7,8,13,20,21 | F |
| *Automolus rufipileatus* | 3,4,7,12,15,17,19,20 | F |
| *Automolus melanopezus* | 7,13 | F |
| *Automolus cervicalis* | 1 | F |
| *Automolus subulatus* | 2,4,5,7,8,13 | F |
| *Automolus ochrolaemus* | 1,2,4,6-8,9-11,12,13,20,22 | F |
| *Automolus infuscatus* | 5-8,10,11-13,18-21 | F |
| *Automolus paraensis* | 2-4,9,14,16,17 | F |
| *Anabazenops dorsalis* | 4,7 | F |
| *Anabacerthia ruficaudata* | 2-4,5,7,9,10,12-14,16-18,20,21 | F |
| *Philydor erythrocercum* | 2-4,5,6,7,9,10,11,13-18 | F |
| *Philydor erythropterum* | 2,4,5,7,13,16 | F |
| *Philydor pyrrhodes* | 1,2-4,7,8,9,10,11-13,17,18,20,21 | F |
| *Syndactyla ucayalae* | 4,7,13 | F |
| *Synallaxis albescens* | 23,25-29,32-34 | S |
| *Synallaxis rutilans* | 1-4,6,7,8,10,12,13,13-17,19,20,22 | F |
| *Synallaxis cherriei* | 4,7 | F |
| *Synallaxis gujanensis* | 27,28 | S |
| *Cranioleuca gutturata* | 7,12,17 | F |
| **Pipridae** |  |  |
| *Neopelma pallescens* | 29,34 | F,S |
| *Neopelma chrysocephalum* | 8 | F |
| *Neopelma sulphureiventer* | 7,12,13 | F |
| *Tyranneutes stolzmanni* | 2-4,5,6,7,8,9-10,13-17,19,20,21 | F |
| *Tyranneutes virescens* | 11,18 | F |
| *Pipra aureola* | 18 | F |
| *Pipra filicauda* | 7,19,22 | F |
| *Pipra fasciicauda* | 4,7,12,13 | F |
| *Ceratopipra erythrocephala* | 1,8,11,18-21 | F |
| *Ceratopipra rubrocapilla* | 2-4,5-7,9,12-17 | F |
| *Ceratopipra chloromeros* | 7 | F |
| *Lepidothrix coronata* | 7,8,13,19,21 | F |
| *Lepidothrix nattereri* | 2,4,6,9 | F |
| *Lepidothrix iris* | 3,16,17 | F |
| *Lepidothrix serena* | 11,18 | F |
| *Manacus manacus* | 1,7,10,14-21 | F |
| *Heterocercus linteatus* | 9 | F |
| *Machaeropterus striolatus* | 7 | F |
| *Machaeropterus pyrocephalus* | 4,7,13,18,19 | F |
| *Dixiphia pipra* | 1,2-4,7,8,10,11,14-21,22 | F |
| *Corapipo gutturalis* | 11,21 | F |
| *Chiroxiphia pareola* | 2,4,6,7-8,10,13-18,19,20 | F |
| **Oxyruncidae** |  |  |
| *Oxyruncus cristatus* | 16,17 | F |
| **Onychorhynchidae** |  |  |
| *Onychorhynchus coronatus* | 1,2-4,6,7,8,9,10,11,12-14,16-18,20,21,22 | F |
| *Terenotriccus erythrurus* | 1,2-4,5,6,7,8,10,11-14,16-20,21 | F |
| *Myiobius barbatus* | 4,6,7,8,10,11,14-17,19,20,21,22 | F |
| *Myiobius atricaudus* | 2,7,19 | F |
| **Tityridae** |  |  |
| *Schiffornis major* | 8 | F |
| *Schiffornis turdina* | 2-4,5,8,9-11,14-17,20,21,22 | F |
| *Schiffornis olivacea* | 1,18 | F |
| *Schiffornis amazonum* | 6,7 | F |
| *Laniocera hypopyrra* | 2,4,5,6,7,8,10,11,14,16,18-20,21,22 | F |
| *Iodopleura isabellae* | 2,3,5,7,10,16,17 | F |
| *Iodopleura fusca* | 19,20 | F |
| *Tityra inquisitor* | 1,3,4,7,10,13,14,16,17,19,20 | F |
| *Tityra cayana* | 1,3,4,5-7,8,11,12-14,17,19-21,22,25-28,34 | F,S |
| *Tityra semifasciata* | 3,4,7,8,10,13-16,18 | F |
| *Pachyramphus viridis* | 15,16 | F |
| *Pachyramphus rufus* | 9,14-17,19,20,22 | F |
| *Pachyramphus castaneus* | 4,5,7,8,13,16 | F |
| *Pachyramphus polychopterus* | 3,6,7,8,13,14,17,19-21,27,28 | F,S |
| *Pachyramphus marginatus* | 1,2-4,8,9,10,11,12-14,16-18,20 | F |
| *Pachyramphus surinamus* | 1,8,11,18,22 | F |
| *Pachyramphus minor* | 1,2,4-6,7,10,11,12,13,16-18,20,21 | F |
| *Pachyramphus validus* | 3,17 | F |
| **Cotingidae** |  |  |
| *Phoenicircus carnifex* | 10,11,17,18 | F |
| *Phoenicircus nigricollis* | 2,4,5,6,8 | F |
| *Haematoderus* *militaris* | 2,3,11,16 | F |
| *Querula purpurata* | 1,6,7,10,12-20 | F |
| *Perissocephalus tricolor* | 1,12,18,19,20,21,22 | F |
| *Cephalopterus ornatus* | 7,19 | F |
| *Lipaugus vociferans* | 2 to 22 | F |
| *Procnias albus* | 1,20 | F |
| *Cotinga maynana* | 7 | F |
| *Cotinga cayana* | 1,2-6,7,8,10,11,13,14,16-18,20,21,22 | F |
| *Cotinga cotinga* | 2,3,16 | F |
| *Porphyrolaema porphyrolaema* | 4,7 | F |
| *Gymnoderus foetidus* | 3,5,7,12,13,18,19 | F |
| *Conioptilon mcilhennyi* | 7,12,13 | F |
| *Xipholena punicea* | 1,2,4,5,6,8,11,18,20,21,22 | F |
| *Xipholena lamellipennis* | 3,10,14,15,17 | F |
| **Pipritidae** |  |  |
| *Piprites chloris* | 1,2-6,7,8,9,10,11,12-18,20,21,22 | F |
| **Platyrinchidae** |  |  |
| *Neopipo cinnamomea* | 2,11 | F |
| *Platyrinchus saturatus* | 1,2-4,10,11,14-18,20,21,22 | F |
| *Platyrinchus coronatus* | 2,4-6,7-8,10,11,13,17,18,20 | F |
| *Platyrinchus platyrhynchos* | 1,2-6,7,8,10,11,12-18,20,21 | F |
| **Rhynchocyclidae** |  |  |
| *Taeniotriccus andrei* | 3,4,14 | F |
| *Cnipodectes subbrunneus* | 5,7,8,12 | F |
| *Cnipodectes superrufus* | 13 | F |
| *Mionectes oleagineus* | 1-6,7,8,9,10,13-15,18-20,21 | F |
| *Mionectes macconnelli* | 3,8,10,11,14,17,18,21,22 | F |
| *Leptopogon amaurocephalus* | 2,4,6,7,12,13,18 | F |
| *Corythopis torquatus* | 2,4,6,7,8,9,11,12,13,16,17,18,20,21,22,34 | F,S |
| *Phylloscartes virescens* | 11,17 | F |
| *Rhynchocyclus olivaceus* | 2,4,5,7,10,11,12,14,18,22 | F |
| *Tolmomyias sulphurescens* | 1,3,4,7,10,14,15,17,19-21 | F |
| *Tolmomyias assimilis* | 1,2,4-6,8,10,11,15-18,20,21 | F |
| *Tolmomyias poliocephalus* | 1,2,4-6,7,11,13,15-18,19,20,22 | F |
| *Tolmomyias flaviventris* | 2,3,7,9,13-15,17,19,20,22-24,27,30,33,34 | F,S |
| *Todirostrum maculatum* | 3,7,9,13,14,19,22,25,27 | F,S |
| *Todirostrum cinereum* | 3,19,25-27,32,33 | F,S |
| *Todirostrum pictum* | 1,11,18,19,20 | F |
| *Todirostrum chrysocrotaphum* | 2-5,7,8,9,13,15 | F |
| *Poecilotriccus albifacies* | 7 | F |
| *Poecilotriccus fumifrons* | 3,18 | F |
| *Myiornis ecaudatus* | 1,2-4,7-8,9,10,11,13-18,19,20,21 | F |
| *Hemitriccus minor* | 2,4,5,6,9,13 | F |
| *Hemitriccus flammulatus* | 6,12,13 | F |
| *Hemitriccus zosterops* | 1,8,11,18,22 | F |
| *Hemitriccus griseipectus* | 4,5,7,13 | F |
| *Hemitriccus iohannis* | 7 | F |
| *Hemitriccus striaticollis* | 33 | S |
| *Hemitriccus margaritaceiventer* | 33,34 | S |
| *Hemitriccus minimus* | 2,4,10,13,16 | F |
| *Lophotriccus vitiosus* | 7,11,18 | F |
| *Lophotriccus eulophotes* | 7,12,13 | F |
| *Lophotriccus galeatus* | 1,3,4,10,11,14,15,17-21,22 | F |
| **Tyrannidae** |  |  |
| *Zimmerius acer* | 1 | F |
| *Zimmerius gracilipes* | 2,5,6,7,8,9,10,11,12,13,15-18,20,21,22 | F |
| *Inezia subflava* | 19,22,24,27 | F,S |
| *Euscarthmus rufomarginatus* | 23,34 | S |
| *Ornithion inerme* | 1,2-4,7,8,9-10,12-18,19,20,20 | F |
| *Camptostoma obsoletum* | 7,9,13-15,17,18,19,22,25,27,30-32,34 | F,S |
| *Elaenia flavogaster* | 14,15,17,19,21,22-25,27-34 | F,S |
| *Elaenia spectabilis* | 7,17,34 | F,S |
| *Elaenia parvirostris* | 2,7,13,21,27,28,31 | F,S |
| *Elaenia cristata* | 25,27,29-34 | S |
| *Elaenia chiriquensis* | 23,24,27,28,30-34 | S |
| *Elaenia ruficeps* | 26,34 | S |
| *Suiriri suiriri* | 23,29,31,32 | S |
| *Suiriri affinis* | 33 | S |
| *Myiopagis gaimardii* | 1,2-4,5,6,7,8,9-11,13-18,19-22 | F |
| *Myiopagis caniceps* | 1,2,4,5,7,8,9,11,13,15-17 | F |
| *Myiopagis viridicata* | 2 | F |
| *Tyrannulus elatus* | 1,2,4,5,7,8,9,11,13-17,19,27,28,31,34 | F,S |
| *Capsiempis flaveola* | 20,21 | F |
| *Phaeomyias murina* | 3,7,9,13-16,19,21,22-28,31,32,34 | F,S |
| *Phyllomyias griseiceps* | 19,21 | F |
| *Polystictus pectoralis* | 24,27 | S |
| *Attila citriniventris* | 2,7 | F |
| *Attila bolivianus* | 7,12 | F |
| *Attila spadiceus* | 1-4,5,7,8,9-12,14-20,21,22 | F |
| *Legatus leucophaius* | 3,4,9,11,14,18,19,20,22,31,34 | F,S |
| *Ramphotrigon megacephalum* | 4,5,7,12 | F |
| *Ramphotrigon ruficauda* | 1-5,7,8,10,11,14,15,17,18,19-21 | F |
| *Ramphotrigon fuscicauda* | 4,7,12 | F |
| *Myiarchus tuberculifer* | 12-4,9,10,11,13,15-18,19,20,21 | F |
| *Myiarchus ferox* | 3,14,15,17,18,19,20,21,22,23-25,27,28,30-32,34 | F,S |
| *Myiarchus tyrannulus* | 22,23-27,30-32,34 | F,S |
| *Sirystes sibilator* | 1,2,4,5,7,11,13,15-18,20 | F |
| *Rhytipterna simplex* | 1,2,4-6,7,8,10,11-18,19-21 | F |
| *Casiornis rufus* | 34 | S |
| *Casiornis fuscus* | 32 | S |
| *Pitangus sulphuratus* | 1,6,13-18,19,29,21,22,24-28,30-34 | F,S |
| *Myiodynastes maculatus* | 8,13,14,16-18,19-21,22,24,31,32,34 | F,S |
| *Tyrannopsis sulphurea* | 1,8,9,1 | F |
| *Megarynchus pitangua* | 4,6,9,12-16,18-20,21,22,23,25,28,31-34 | F,S |
| *Myiozetetes cayanensis* | 6,13-21,22,24,25,30,31 | F,S |
| *Myiozetetes similis* | 18,19,28,32,34 | F,S |
| *Myiozetetes luteiventris* | 1,2,4,9,10,13,20 | F |
| *Tyrannus melancholicus* | 9,14-21,22-25,27,28,30-34 | F,S |
| *Empidonomus varius* | 4,6,9,14-17,19-21,22,25,27,28,31,32,34 | F,S |
| *Conopias trivirgatus* | 7,19,32 | F,S |
| *Conopias parvus* | 1,2,5,7,8,9,11,17,19,20 | F |
| *Myiophobus fasciatus* | 19,28 | F,S |
| *Sublegatus modestus* | 25,32,33 | S |
| *Pyrocephalus rubinus* | 24,25,27,32,34 | S |
| *Fluvicola pica* | 24,26,27,34 | S |
| *Fluvicola albiventer* | 34 | S |
| *Cnemotriccus fuscatus* | 6,7,24-27,34 | F,S |
| *Lathrotriccus euleri* | 1,3,4,7,9,13,15,16,19,30,34 | F,S |
| *Contopus nigrescens* | 3,10 | F |
| *Xolmis cinereus* | 23,29,30,33 | S |
| **Passeri** |  |  |
| **Vireonidae** |  |  |
| *Cyclarhis gujanensis* | 1,4,7,9,11,13,14,17-22,-28,30,31,32 | F,S |
| *Vireolanius leucotis* | 2,4,5,7,8,9,10,11-13,18,19,20 | F |
| *Hylophilus pectoralis* | 14,17,19,21 | F |
| *Hylophilus semicinereus* | 3,4,9-10,14,15,17 | F |
| *Hylophilus thoracicus* | 1,7,8,13,18 | F |
| *Tunchiornis ochraceiceps* | 1,2,4,5,6,7,8,9,10,11,13,15-18,20,21,22 | F |
| *Pachysylvia muscicapina* | 1,2,11,18,19,20,21 | F |
| *Vireo olivaceus* | 3,4,7,9,10,13,18-21,23,25,32 | F,S |
| **Corvidae** |  |  |
| *Cyanocorax violaceus* | 7,19,20,21 | F |
| *Cyanocorax cristatellus* | 34 | S |
| *Cyanocorax cayanus* | 18,21 | F |
| **Hirundinidae** |  |  |
| *Stelgidopteryx ruficollis* | 27-29,31,33,34 | S |
| *Progne tapera* | 25,31,32 | S |
| *Progne chalybea* | 25,29,32-34 | S |
| **Troglodytidae** |  |  |
| *Microcerculus marginatus* | 2,4,5,6,7,9,10,12-17 | F |
| *Microcerculus bambla* | 8,11,19,21,22 | F |
| *Odontorchilus cinereus* | 2,4,10 | F |
| *Troglodytes musculus* | 23-32,34 | S |
| *Campylorhynchus griseus* | 25,27 | S |
| *Campylorhynchus turdinus* | 4,6,7,10,13 | F |
| *Pheugopedius genibarbis* | 2-4,7,12-17 | F |
| *Pheugopedius coraya* | 1,8,10,11,18-20,21,22 | F |
| *Cantorchilus leucotis* | 3,7,14,19,20,24,33 | F,S |
| *Henicorhina leucosticta* | 21 | F |
| *Cyphorhinus arada* | 2-8,10,11,18,19,22 | F |
| **Polioptilidae** |  |  |
| *Microbates collaris* | 1,8,11,18,19,20,21,22 | F |
| *Ramphocaenus melanurus* | 1,2-4,5,6,7,8,9,10,12-17,19,20,21 | F |
| *Polioptila plumbea* | 7,12,14,15,18-20,22,24-27 | F,S |
| *Polioptila guianensis* | 4,5,10,11,17,20 | F |
| *Polioptila paraensis* | 2 | F |
| *Polioptila facilis* | 8 | F |
| *Polioptila dumicola* | 33 | S |
| **Turdidae** |  |  |
| *Turdus leucomelas* | 14,16,18,19,21,23,25,30-33 | F,S |
| *Turdus fumigatus* | 1,3,6,14,15,19,20 | F |
| *Turdus hauxwelli* | 2,7,8,12-17 | F |
| *Turdus nudigenis* | 3,19 | F |
| *Turdus lawrencii* | 2,4,6,7 | F |
| *Turdus ignobilis* | 30,32 | S |
| *Turdus albicollis* | 1-6,7-10,11,13-21,22 | F |
| **Mimidae** |  |  |
| *Mimus gilvus* | 25-28 | S |
| *Mimus saturninus* | 23,29,30,32-34 | S |
| **Motacillidae** |  |  |
| *Anthus lutescens* | 25,27,29,34 | S |
| **Passerellidae** |  |  |
| *Zonotrichia capensis* | 28,30,32,34 | S |
| *Ammodramus humeralis* | 23-32, 34 | S |
| *Arremonops conirostris* | 19,27 | F,S |
| *Arremon taciturnus* | 1,2,4,7,8,8,11,12,13-21,22,33 | F,S |
| **Parulidae** |  |  |
| *Basileuterus culicivorus* | 4,21,34 | F,S |
| *Myiothlypis flaveola* | 34 | S |
| *Myiothlypis fulvicauda* | 6,7,13 | F |
| *Myiothlypis mesoleuca* | 3,10,11,14,15,17,18,19-21,22 | F |
| **Icteridae** |  |  |
| *Psarocolius angustifrons* | 7,12 | F |
| *Psarocolius viridis* | 1,2,5,6,7,8,10-11,14,17-20,21,22 | F |
| *Psarocolius decumanus* | 1,3,6,7,9,12-15,21,22,32 | F,S |
| *Psarocolius bifasciatus* | 1,2-4,7,9,10,12-16 | F |
| *Procacicus solitarius* | 3,7,16,19 | F |
| *Cacicus haemorrhous* | 1,2-4,7,8,10,11,14,16,18,19,20,21 | F |
| *Cacicus oseryi* | 7 | F |
| *Cacicus cela* | 1,3,4,6,7,9,12-17,19-22,25,32,33 | F,S |
| *Icterus cayanensis* | 2-4,7,14-16,18 | F |
| *Icterus chrysocephalus* | 1,7,8,11,19-21,22 | F |
| *Icterus nigrogularis* | 24-27 | S |
| *Icterus croconotus* | 24 | S |
| *Molothrus oryzivorus* | 30,31 | S |
| *Molothrus bonariensis* | 23-25,27,32 | S |
| *Sturnella militaris* | 23-28,30-32 | S |
| *Sturnella magna* | 23,25-28,30 | S |
| **Mitrospingidae** |  |  |
| *Lamprospiza melanoleuca* | 2-6,7,9,10,11-17 | F |
| **Thraupidae** |  |  |
| *Parkerthraustes humeralis* | 2-4,7,10 | F |
| *Neothraupis fasciata* | 23 | S |
| *Cissopis leverianus* | 4,14,19-21 | F |
| *Schistochlamys melanopis* | 4,14,19,23,28,30-34 | F,S |
| *Schistochlamys ruficapillus* | 34 | S |
| *Tangara gyrola* | 2,4,7,12,14,21,22 | F |
| *Tangara schrankii* | 2,4,5,7,12,13 | F |
| *Tangara mexicana* | 1,3,4,5,6,7,8,9,10,11,12,14,15,19,20,22 | F |
| *Tangara chilensis* | 2,4-9,11,12,13,15,19,20,21 | F |
| *Tangara velia* | 1,2-4,5,7,8,10,11,13,15-17,19,20,21 | F |
| *Tangara callophrys* | 7,12,13 | F |
| *Tangara varia* | 22 | F |
| *Tangara punctata* | 1,4,8,10,11,14,20,21 | F |
| *Tangara xanthogastra* | 73,19,21 | F |
| *Tangara episcopus* | 1,3,14-21,22,25,27,28,30-32,34 | F,S |
| *Tangara palmarum* | 1,3,14-21,22,23,25-28,30,32,34 | F,S |
| *Tangara nigrocincta* | 4,7,13,20,21 | F |
| *Tangara cayana* | 3,6,18,19,21,22,25,29-34 | F,S |
| *Nemosia pileata* | 4,7,19,20,22,24,25,29,31,32 | F,S |
| *Cyanicterus cyanicterus* | 5,20 | F |
| *Conirostrum speciosum* | 22 | F |
| *Sicalis citrina* | 28,32,34 | S |
| *Sicalis luteola* | 24-28,30,32 | S |
| *Chlorophanes spiza* | 1-5,7,8,10,11,12-17,19-21 | F |
| *Hemithraupis flavicollis* | 2,4,5,7,8,11,13,18,19,20,21,33 | F,S |
| *Hemithraupis guira* | 3,6,7,12-17,19-21,27,28,34 | F,S |
| *Volatinia jacarina* | 23,24,27,28,30-32,34 | S |
| *Eucometis penicillata* | 1,7,20 | F |
| *Lanio surinamus* | 1,2,6,7,8,10,11,12,14,15,17-20,21 | F |
| *Lanio versicolor* | 2,4,5,7,10,12-14 | F |
| *Lanio fulvus* | 11,18,20 | F |
| *Lanio luctuosus* | 1,2,4,7,10,12,13,19,20 | F |
| *Lanio cristatus* | 2,4,5,6,7,8-11,13-20,21,22 | F |
| *Tachyphonus phoenicius* | 28 | S |
| *Tachyphonus rufus* | 3,14-16,18,31,32,34 | F,S |
| *Ramphocelus carbo* | 3,14-17,19-21,22,23,25,30,34 | F,S |
| *Tersina viridis* | 4,6,7,15,19,21,22,31 | F,S |
| *Cyanerpes nitidus* | 1,2,4,5,7,8,11,17,20 | F |
| *Cyanerpes caeruleus* | 2-4,5,6,7,8,10,12,14-17,19-21 | F |
| *Cyanerpes cyaneus* | 1,2-4,5,7,8,10,11,16,18,19,21,22 | F |
| *Dacnis albiventris* | 4 | F |
| *Dacnis flaviventer* | 4,5,7,12,18 | F |
| *Dacnis cayana* | 1,2,4,5-7,8,11,13-15,17-21,22,25-28,30-33 | F,S |
| *Dacnis lineata* | 3,4,5-7,10,11,13,14,15,19,20,21 | F |
| *Coereba flaveola* | 2,4,6,11,14-18,19,21,22,24,25,27,28,30,32,33,34 | F,S |
| *Sporophila lineola* | 28,33,34 | S |
| *Sporophila schistacea* | 19,20 | F |
| *Sporophila intermedia* | 24,25,27 | S |
| *Sporophila plumbea* | 23-25,27,28,30,32,34 | S |
| *Sporophila americana* | 27,30 | S |
| *Sporophila nigricollis* | 25,28,32 | S |
| *Sporophila leucoptera* | 25,29 | S |
| *Sporophila bouvreuil* | 29 | S |
| *Sporophila minuta* | 24,27,28 | S |
| *Sporophila angolensis* | 23,24,25,27,28,30-34 | S |
| *Emberizoides herbicola* | 23,24,28-30 | S |
| *Saltator maximus* | 3,4,6,9,11,13-20,21,22,33 | F,S |
| *Saltator coerulescens* | 6,9,13,19,21,26,27,28 | F,S |
| *Saltator grossus* | 1,2,4,7,8,9-20 | F |
| *Cypsnagra hirundinacea* | 23,29,30 | S |
| **Cardinalidae** |  |  |
| *Piranga flava* | 23,25,27,29,31,32 | S |
| *Habia rubica* | 2,4,5,6,7,8,9,10,13,18 | F |
| *Granatellus pelzelni* | 1,2-4,9,10,15-18,20,21 | F |
| *Caryothraustes canadensis* | 3,8,9,11,14-17,20,21,28 | F,S |
| *Periporphyrus erythromelas* | 10,14-16 | F |
| *Cyanoloxia rothschildii* | 1,4,5,6,7-10,11-15,17-20,21 | F |
| **Fringillidae** |  |  |
| *Spinus magellanicus* | 28 | S |
| *Euphonia plumbea* | 21 | F |
| *Euphonia chlorotica* | 3,14,19,22,23,30-34 | F,S |
| *Euphonia finschi* | 19,22 | F |
| *Euphonia violacea* | 1,3,4,10,15,18,19-21 | F |
| *Euphonia laniirostris* | 5,6,7,13 | F |
| *Euphonia chrysopasta* | 1,2,4,5,7,8,11,16-18,19,20 | F |
| *Euphonia minuta* | 1,2,4,7,10,11,13,14,20,21 | F |
| *Euphonia xanthogaster* | 2,4,6,7,19 | F |
| *Euphonia rufiventris* | 2,4,5,7,8,9,10,13,20 | F |
| *Euphonia cayennensis* | 1,3,11,14-18,21 | F |

| **Appendix S3** Birds species in forest and savanna sites in the Amazon and that are absent from the BirdTree phylogeny. These species were inserted as polytomies in the phylogenetic tree used in this study. Species indicated with asterisks (*) constitute splits and those indicated with the number (¹) constitute new descriptions for science. | | |
| --- | --- | --- |
| **Bird families** | **Absent from BirdTree** | **Inserted as politomy from...** |
| Dendrocolaptidae | *Dendrocolaptes medius** | *Dendrocolaptes certhia* |
| Dendrocolaptidae | *Lepidocolaptes duidae** | *Lepidocolaptes albolineatus* |
| Dendrocolaptidae | *Lepidocolaptes layardi** | *Lepidocolaptes albolineatus* |
| Dendrocolaptidae | *Xiphorhynchus chunchotambo** | *Xiphorhynchus ocellatus* |
| Furnariidae | *Automolus cervicalis** | *Automolus infuscatus* |
| Furnariidae | *Automolus paraensis** | *Automolus infuscatus* |
| Grallariidae | *Hylopezus paraensis** | *Hylopezus macularius* |
| Icteridae | *Icterus chrysocephalus* | *Icterus cayanensis* |
| Parulidae | *Myiothlypis mesoleuca* | *Phaeothlypis rivularis* e *P.* *fulvicauda* |
| Pipridae | *Machaeropterus striolatus* | *Machaeropterus regulus* |
| Polioptilidae | *Polioptila paraensis* | *Polioptila guianensis* e *P*. *facilis* |
| Polioptilidae | *Polioptila facilis* | *Polioptila guianensis* |
| Thamnophilidae | *Epinecrophylla pyrrhonota* | *Epinecrophylla haematonota* |
| Thamnophilidae | *Herpsilochmus stotzi¹* | *Herpsilochmus atricapillus* |
| Thamnophilidae | *Percnostola subcristata* | *Percnostola rufifrons* |
| Thamnophilidae | *Percnostola minor* | *Percnostola rufifrons* |
| Thamnophilidae | *Willisornis vidua** | *Willisornis poecilinotus* |
| Tytiridae | *Schiffornis amazonum* | *Schiffornis turdina* |
| Tytiridae | *Schiffornis olivacea* | *Schiffornis turdina* |
| Dendrocolaptidae | *Campylorhamphus probatus* | *Campylorhamphus procurvoides* |
| Troglodytidae | *Troglodytes musculus** | *Troglodytes aedon* |
